# Targeting the DNA Damage Response for the Treatment of High Risk Neuroblastoma

**DOI:** 10.3389/fonc.2020.00371

**Published:** 2020-04-03

**Authors:** Harriet E. D. Southgate, Lindi Chen, Nicola J. Curtin, Deborah A. Tweddle

**Affiliations:** ^1^Wolfson Childhood Cancer Research Centre, Newcastle University Centre for Cancer, Translational and Clinical Research Institute, Faculty of Medical Sciences, Newcastle University, Newcastle upon Tyne, United Kingdom; ^2^Newcastle University Centre for Cancer, Translational and Clinical Research Institute, Faculty of Medical Sciences, Newcastle University, Newcastle upon Tyne, United Kingdom

**Keywords:** neuroblastoma, DNA damage response, targeted therapy, PARP, ATR

## Abstract

Despite intensive multimodal therapy, the survival rate for high risk neuroblastoma (HR-NB) remains <50%. Most cases initially respond to treatment but almost half will subsequently relapse with aggressive treatment resistant disease. Novel treatments exploiting the molecular pathology of NB and/or overcoming resistance to current genotoxic therapies are needed before survival rates can significantly improve. DNA damage response (DDR) defects are frequently observed in HR-NB including allelic deletion and loss of function mutations in key DDR genes, oncogene induced replication stress and cell cycle checkpoint dysfunction. Exploiting defects in the DDR has been a successful treatment strategy in some adult cancers. Here we review the genetic features of HR-NB which lead to DDR defects and the emerging molecular targeting agents to exploit them.

## Introduction

Neuroblastoma (NB) is a rare childhood cancer derived from cells of the embryonal neural crest. Many cancers, including NB, show a high number of chromosomal genetic abnormities, including rearrangement of chromosomes or gains and losses of whole or parts of chromosomes and less frequently changes to the nucleic acid sequence. This dynamic process is known as genome instability and is described as an enabling characteristic allowing cancer cells to acquire six major hallmarks required for survival and proliferation, first outlined by Hanahan and Weinburg in 2000 and updated in 2011. These hallmarks include self-sufficiency in growth signals, insensitivity to anti-growth signals, infinite replication, evasion of apoptosis, angiogenesis, and tissue invasion and metastasis (1, 2).

Genomic instability can arise due to defects in the DNA damage response (DDR) (3). The DDR is a highly orchestrated network which signals DNA damage to cell cycle checkpoints (G_1_/S, intra-S and G_2_/M) resulting in cell cycle arrest (4). Dysregulation of cell cycle control allows for mutations to accumulate. The combination of genetic instability and loss of cell cycle control results in a “mutator phenotype” in which mutations are frequently established and maintained (5). Loss of G_1_ checkpoint control, through mutations in the *TP53* or *RB* tumor suppressor genes, activation of oncogenes such as *Ras* or *MYC*, or imbalance in G_1_/S cyclins, cyclin-dependent kinases (CDKs) and their inhibitors, is a common feature of cancer cells (6), making these cells dependent on the G_2_ checkpoint for survival after DNA-damaging treatments.

Another cause of chromosome instability is DNA replication stress (7), a state in which the DNA replication machinery cannot maintain the rate of DNA synthesis resulting in increased replication fork stalling and collapse (8, 9). Replication stress is common in NB, and many other cancers, due to the overexpression of oncogenes driving rapid proliferation and loss of G_1_ checkpoint control, and provides an exploitable cancer-specific defect. Targeting the DDR can not only exploit cancer-specific defects, but could also overcome resistance to cytotoxic chemo- and radiotherapy resulting from upregulation of DNA repair pathways in cancer (10).

## Neuroblastoma

NB is the commonest extra-cranial malignant solid tumor of infancy and accounts for 8% of all childhood (0–14 years) cancers in the UK (11). Around 100 new cases are diagnosed each year in the UK. Tumors usually appear in very young children, the median age of diagnosis being 17 months (12). NB is a neuroendocrine tumor derived from precursor cells of the sympathetic nervous system resulting in tumors in the adrenal glands or sympathetic ganglia (13). Most NB tumors present in the abdomen but can also appear in the neck, chest or pelvis in paraspinal regions. Tumors are highly heterogeneous both phenotypically and clinically, with outcome varying from maturation or spontaneous regression to aggressive progression (14). In addition to a variety of molecular markers associated with outcome (discussed in section Genetics of Neuroblastoma), studies have shown that the degree of tumor cell differentiation is related to patient survival (15). Pathologically, tumors show varying degrees of differentiation from NB which is predominantly composed of undifferentiated or poorly differentiated small round tumor cells to ganglioneuroblastoma intermixed, which consists of both immature cells and terminally differentiated ganglion cells to a mature ganglioneuroma (13). Tumors showing a higher degree of cell differentiation usually have a better prognosis than undifferentiated tumors. Tumor differentiation, age at diagnosis, tumor stage and molecular abnormalities are variables used to classify NB into risk groups which define treatment strategies (discussed in section Neuroblastoma Risk Stratification).

### Genetics of Neuroblastoma

#### *MYCN* Amplification

Amplification of the *MYCN* oncogene, either as intra-chromosomal homogenously staining regions (HSRs) or as extrachromosomal double minutes (16), is seen in around 20% of all NB cases and is one of the strongest unfavorable prognostic markers (17). The frequency of *MYCN* amplification increases to around 50% in the high-risk group (18). *MYCN* is a member of the *MYC* family of proto-oncogenes which also includes c-*MYC* and *MYCL* (19–21). The *MYC* family of proteins are basic-helix–loop–helix-leucine zipper (bHLH-LZ) transcription factors which mediate mitogen signaling by regulating transcription of target genes involved in metabolism, protein biosynthesis, cell cycle regulation, DNA repair, cell adhesion, and the cytoskeleton (22, 23). They therefore have a critical role in cellular proliferation, differentiation, apoptosis, and oncogenesis. In contrast to *c-MYC*, which is expressed in a variety of embryonal and adult tissues, expression of *MYCN* is restricted to the developing nervous system and only a few other sites (24–26). Ectopic expression of *MYCN* drives cell proliferation but also leads to sensitization to apoptosis through activation of the tumor suppressor protein p53 (27), therefore mechanisms to evade MYCN induced apoptosis are essential for NB development [reviewed by (28)]. This may be achieved by loss of expression of the initiator caspase, caspase 8 (29–31), which mediates the extrinsic death receptor apoptosis pathway (32, 33). A functional MYCN/c-MYC signature also characterizes a fraction of aggressive NB without *MYCN* amplification (34, 35), which suggests that increased MYC activity is a main driver of aggressiveness in neuroblastoma.

Increased expression of *MYC* oncogenes drives rapid, erroneous replication leading to replication stress (36).

#### Segmental Chromosome Alterations

Many diploid and tetraploid NB tumors show numerous non-random structural chromosome alterations, such as deletion of chromosomes 1p, 3p, 4p, 11q, and gain of 1q, 2p, and 17q, which are associated with poor prognosis (37–39). Gain of chromosome 17q and loss of chromosome 1p are observed in half and a third of NB cases, respectively, and correlate with *MYCN* amplification and poor prognosis (40, 41). 11q loss is also observed in about third of NB tumors and is a marker of poor prognosis independent of *MYCN* status (discussed in section 11q Loss) (40). Chromosome 2p is the location of both the *MYCN* and *ALK* genes (discussed in section ALK and MAP Kinase Pathways) (42), therefore gain of 2p could contribute to overexpression of both of these genes. In general, the presence of structural chromosome alterations, in contrast to whole chromosome gains or losses (numerical chromosome alterations), is associated with advanced stage of disease and inferior outcome due to the former being associated with genomic instability whereas the latter is associated with mitotic defects (38).

#### 11q Loss

**Figure 1:**
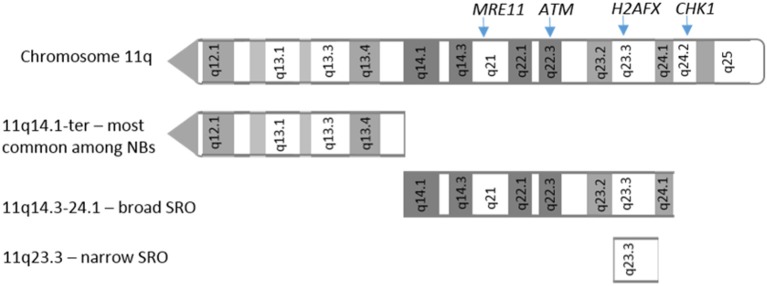
11q deletions in neuroblastoma. Adapted from Mlakar et al. (44). Location of 11q arm deletions observed in neuroblastoma tumors. SRO: shortest region of overlap.

A common structural chromosome aberration is 11q loss, which is seen in around 30–40% of NBs. Many high risk, non-*MYCN* amplified NB tumors show 11q deletion. *MYCN* amplification and 11q loss rarely occur together, suggesting a degree of mutual exclusivity. The smallest region of overlap in 11q deletions has been reported between 11q14 and 11q23 (43) including genes such as *CADM1* (11q23.3), and 4 genes involved in the DDR: *ATM* (11q22.3), *CHK1* (11q24.2), *MRE11* (11q21), and *H2AFX* (11q23.3), which have been functionally tested as candidate genes responsible for driving NB tumorigenesis [Figure 1; (44)]. No mutation or hyper-methylation was found in the other allele of these genes in most cases (44), however loss of one copy via 11q deletion could contribute to tumorigenesis due to haploinsufficiency.

Homozygous germline mutations in *ATM* (ataxia-telangiectasia mutated) cause ataxia telangiectasia (A-T), a recessive genetic disease characterized by cerebellar degeneration, chromosomal instability and cancer predisposition. ATM is a key DDR protein which signals to DNA repair machinery and leads to cell cycle arrest in response to double strand DNA breaks (DSBs) [reviewed in (45)]. Furthermore, somatic mutations of *ATM* have been identified in many cancer types, most commonly lymphoid malignancies (46), suggesting that ATM loss contributes to tumorigenesis. ATM deficiency results in impaired cell cycle arrest through disrupted signaling to p53 (discussed section p53 Pathway) (47, 48), enabling mutations to accumulate in the genome.

*CHK1, MRE11*, and *H2FAX* also encode proteins integral to the DDR. Chk1 is the primary target of ataxia-telangiectasia and rad3-related (ATR) kinase which, similar to ATM, signals to DNA repair and cell cycle checkpoints in response to DNA damage. Mre11 is part of the Mre11-Rad50-Nbls1 (MRN) complex, a multiprotein complex which regulates repair of DSBs. In contrast to *ATM, Mre11* is essential for cell survival and homozygous hypomorphic *Mre11* mutations lead to a genetic disease which is phenotypically similar to A-T called ataxia telangiectasia-like disorder (49). *H2AFX* encodes a variant of histone 2A which can be phosphorylated. H2AX is phosphorylated in response to DSB formation by DDR kinases, such as ATM, which results in the recruitment of DNA repair machinery and chromatin remodeling complexes and the amplification of the signal by spreading along chromatin (50). H2AX deficiency leads to chromosome instability.

Overall, heterozygous loss of 11q results in loss of DDR proteins which leads to chromosome instability allowing cancer to develop.

#### p53 Pathway

The tumor suppressor protein p53 is one of the main downstream targets of the ATM signaling pathway. p53 is a transcription factor that has a crucial role in maintaining genome integrity and tumor suppression and is activated in response to a variety of intra- and extra-cellular stresses, including DNA damage, oncogene activation, oxidative stress, deficient growth factors/signals, ribonucleotide depletion, and hypoxia (51). Activated p53 regulates transcription of genes involved in cell cycle arrest, apoptosis, senescence, DNA repair, differentiation, angiogenesis, metastasis and metabolism (52–54). *TP53*, is the most frequently mutated gene in human cancer (55).

**Figure 2:**
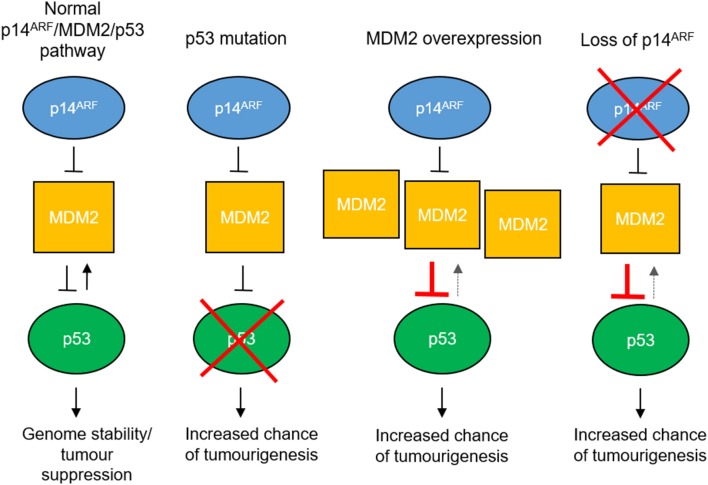
Mechanisms of p53 pathway dysfunction. The p53 pathway can be disrupted in cancer by mutation of the *TP53* gene, overexpression of *MDM2* e.g., by gene amplification or loss of *p14*^*ARF*^ expression by gene methylation or deletion.

Loss of p53 function is also seen in tumors with wild-type p53 (52). Upregulation of MDM2, an E3 ubiquitin ligase which targets p53 for degradation (56), or loss of p14^ARF^ function, which inhibits MDM2 (57, 58), causes decreased p53 stability and reduced p53 function (Figure 2).

In neuroblastoma, *TP53* mutations are rare at diagnosis, however aberrations in the p53 pathway are observed more frequently at relapse (59–62), where around 50% of relapsed cases analyzed show *TP53* mutation, *MDM2* amplification or *p14*^*ARF*^ inactivation, suggesting that p53 inactivation could be a contributor to acquired drug resistance. In addition, *TP53* is located on chromosome 17p13.1 and allelic loss of 17p has been observed in both NB cell lines and tumors (63, 64), more frequently in cell lines derived at relapse (61), indicating that 17p loss could be a mechanism by which p53 function is reduced.

#### Telomere Maintenance

Telomere maintenance is essential for establishment of high risk NB (65). High telomerase expression indicates increased invasiveness and poor prognosis (66), comparable to *MYCN* amplified tumors (67). Rearrangements at the *TERT* gene locus (5p15.33) are frequent in NB resulting in overexpression of the *TERT* gene and subsequent increased telomerase expression (67, 68). In contrast to other cancers, mutations in the *TERT* promoter region are rare in NB primary tumors and cell lines (69) and *TERT* activation is most likely achieved by amplification or juxtapositions of *TERT* to strong enhancer elements (70, 71). *TERT* is known to be a transcriptional target of MYCN (72), and *MYCN* amplified NB cells show increased *TERT* expression in comparison to non-*MYCN* amplified in the absence of *TERT* rearrangements (70).

In high risk NB tumors which do not express telomerase, a recombination mediated mechanism known as alternate lengthening of telomeres (ALT) is activated. ALT activity in NB is associated with mutations in the α-thalassaemia/mental retardation syndrome X-linked (*ATRX*) gene (73–75). Loss of function mutations in *ATRX* are among the most common genetic lesions in NB (73, 76). *ATRX* encodes an RNA-helicase which plays a role in chromatin remodeling, nucleosome assembly and telomere maintenance (77). *ATRX* mutation has been shown to be mutually exclusive with *MYCN* amplification (78) and is often seen in tumors from older patients, such as adolescent or young adult but have also been observed in children over 5 years, and is associated with a chronic or indolent disease course (73). Identifying *ATRX* mutations could define a subset of NB cases in which the ALT pathway could be targeted to improve treatment. Recently, loss of ATRX function has not only been shown to be mutually exclusive with MYCN amplification, but also incompatible with overexpression of the MYCN protein due to intolerable levels of replication stress (79). This suggests potential synthetically lethal approaches that could be explored by targeting ATRX function in MYCN-driven tumors, or inducing MYCN-related metabolic changes in ATRX mutant NB.

#### ALK and MAP Kinase Pathways

Activating mutations in the anaplastic lymphoma kinase (*ALK*) gene have been reported in 50% of familial NB (familial NB is rare accounting for around 2% of NB cases) and between 8 and 10% of sporadic neuroblastoma, across all risk groups and occurring more frequently at relapse (42, 78, 80–82). In neuroblastoma, the constitutive activation of ALK, and subsequent downstream pathways, have been shown to be involved in cell proliferation, inducing replication stress, migration, and invasion (83). As well-mutations, aberrant ALK activity has also been reported through *ALK* amplification (84, 85). *ALK* amplification has been shown to be accompanied by *MYCN* amplification and there is evidence that ALK activation accelerates MYCN driven tumorigenesis in animal models (84, 85). ALK is a receptor tyrosine kinase (RTK) specifically expressed in the developing nervous system (86, 87). Like other RTKs, ligand binding leads to receptor activation by dimerization and auto-phosphorylation, recruitment of adaptor proteins and downstream signal transduction through pathways such as PI3K/AKT, RAS/MAPK, and JAK/STAT (88–90). In addition to *ALK* aberrations, mutations in components of the RAS-ERK/MAPK pathway are frequently observed at relapse and are likely contributors to therapy resistance (91). These mutations include activating mutations in *BRAF, RAS* (*KRAS* and *HRAS*), and *PTPN11* (78), which encodes the tyrosine phosphatase SHP-2, and inactivating mutations in the *NF1* tumor suppressor gene (91), a negative regulator of RAS (92). Activation of the RAS signaling pathway leads to replication stress by driving DNA replication, by a similar mechanism to MYCN overexpression (93, 94).

### Neuroblastoma Risk Stratification

At diagnosis, NB cases are categorized into three risk groups, low, intermediate and high risk according to the International Neuroblastoma risk group (INRG) classification system on the basis of age at diagnosis, tumor stage, histopathology and molecular abnormalities including *MYCN* status and DNA copy number abnormalities (17). The probability of disease free survival for each group is 95–100%, 85–90%, and <50%, respectively (95). High risk NB (HR-NB) accounts for around 50% of all NB cases (17) and, despite intensive multi-modal therapy, only 50% of patients with HR-NB are cured (96). New treatments and a better understanding of drug resistance are needed before these survival rates can significantly improve. Risk category defines which treatment strategy to follow and correlates with outcome, with low risk showing the best outcome and high risk showing the poorest (97).

### Current Treatment of Neuroblastoma

Treatment strategies in NB are defined by risk classification. Low risk disease will often spontaneously regress and generally results in a good outcome with clinical observation or surgical resection alone. For intermediate risk, treatment regimens are response dependent and vary from 4 to 8 cycles of conventional chemotherapy, which is often at lower doses than high risk regimens, and the primary tumor is surgically resected where possible.

HR-NB is currently treated with a number of different DNA damaging agents during induction and consolidation according to the previous European High risk NB trial (HR-NBL1, NCT01704716) including cisplatin, carboplatin, etoposide, vincristine, cyclophosphamide, topotecan and doxorubicin during induction, and high dose busulfan and melphalan myeloablative therapy with autologous stem cell rescue followed by local radiotherapy to the site of the primary tumor during consolidation.

Immunotherapy with the anti-GD_2_ chimeric mono-clonal antibody Dinutuximab was approved in 2015 by the Food and Drug Administration (FDA) for maintenance treatment in combination with GM-CSF and 13-cis retinoic acid for pediatric HR-NB patients (98). Dinutuximab beta was also recommended by the National Institute for Health and Care Excellence (NICE) in 2018 for maintenance treatment of HR-NB.

With these regimens the majority of patients will respond to treatment but over 50% of cases will relapse and very few relapsed patients can then be cured (96, 99). Presently at relapse patients are given a backbone chemotherapy of temozolomide and irinotecan to which new agents are added.

Due to the intensive treatment of high risk disease, surviving patients often suffer from multiple sequelae (100). Selective inhibitors of cancer specific aberrant pathways have the potential to replace these conventional chemotherapeutics or decrease the dose required for therapeutic effect, thus reducing the toxic side effects of HR-NB treatment.

## Targeting the DDR in High Risk Neuroblastoma

The differential response of cancers to current anti-cancer therapies are likely to be dependent on the DNA damage response (DDR). Although some DDR dysfunction enables cancer development and increased therapeutic resistance (101), defects in particular pathways are exploitable with the appropriate conventional therapy or novel agents targeting components of the DDR [reviewed by (102)], selectively killing the cancer cells. Cells from HR-NB tumors show a high degree of chromosome instability in the form of segmental chromosome aberrations, including allelic gains, and losses of chromosomes and regional amplifications (38).

The DDR is a highly orchestrated signaling system which detects DNA damage and signals to cellular responses including cell cycle checkpoint arrest, DNA repair, and apoptosis (103). It has evolved to allow cells to survive high levels of endogenous and environmental DNA damage and prevent damaged DNA being copied and passed on to daughter cells.

### Cell Cycle Checkpoint Signaling and Replication Stress

**Figure 3:**
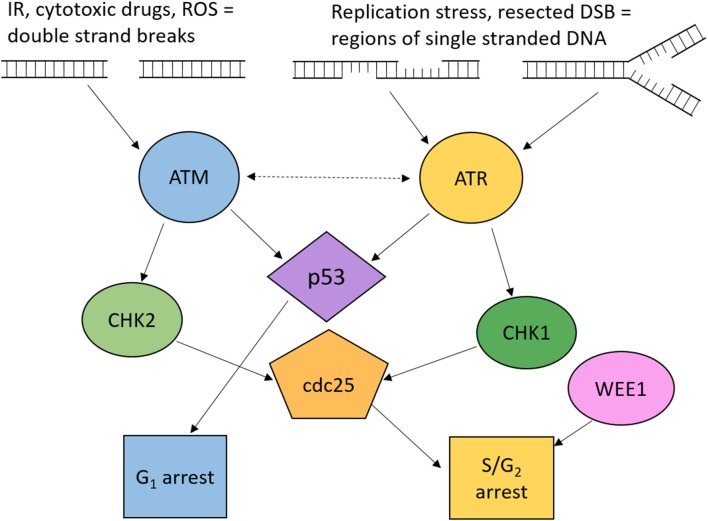
Overview of ATM and ATR signaling to cell cycle checkpoint arrest. Cell cycle arrest is induced through ATM and ATR dependent phosphorylation of p53, CHK1, and CHK2. Active p53 induces G_1_ arrest. Active CHK1 and CHK2 phosphorylate Cdc25 phosphatases resulting in S and G_2_ arrest. Wee1 kinase is also key to maintaining G_2_ cell cycle arrest. ROS, reactive oxygen species; DSB, double strand breaks; IR, ionizing radiation.

DNA damage sensors initiate cell cycle arrest by the activation of downstream signaling pathways. Two of these sensors are ataxia telangiectasia mutated (ATM) and ataxia telangiectasia and Rad3-related (ATR). The primary targets of ATM and ATR are the checkpoint kinases CHK2 and CHK1, respectively, which signal to checkpoint arrest by regulation of the proteins involved in cell cycle progression, demonstrated in Figure 3.

ATM is activated in response to DSBs and plays a crucial role in the activation of the G_1_/S cell cycle checkpoint which is primarily mediated through p53 activity. ATM can also signal to S and G_2_/M checkpoints via CHK2. CHK2 phosphorylates cdc25A, preventing S phase progression, and cdc25C, preventing the transition into mitosis [Figure 3; (104)].

ATR is activated by regions of single-stranded DNA (ssDNA), such as regions at stalled replication forks or formed by resection during DNA repair by homologous recombination repair (HRR) (45). It signals to CHK1 which phosphorylates cdc25A and cdc25C, leading to their inhibition (105). Although many ATR substrates overlap with ATM, loss of ATR is embryonically lethal, whereas loss of ATM is viable, therefore some roles of ATR are essential. One of the essential functions is the role of ATR in survival through replication stress. In addition to inducing cell cycle arrest, ATR prevents replication origin firing, thus reducing the number of active forks, maintains stability of stalled replication forks and promotes replication restart (106).

### DNA Repair

Endogenous DNA damage is repaired by a number of pathways specific to the type of damage sustained. These pathways work together to ensure any damage to DNA is repaired with high fidelity to maintain genome integrity. DNA lesions caused by DNA damaging chemotherapeutic agents are also repaired by these pathways. Table 1 outlines the mechanism by which cytotoxic agents used in the treatment of HR-NB inflict DNA damage and the pathways involved in subsequent DNA repair.

**Table 1:** DNA damaging mechanism of chemotherapeutic agents used in the treatment of high risk NB (107).

**Therapy**	**Mechanism of action**	**Pathways involved in repair**
Cisplatin, carboplatin	Platinum based crosslinking agent	FA pathway including NER and HRR
Cyclophosphamide	Crosslinking agent (nitrogen mustard)	FA pathway including NER and HRR
Etoposide, Doxorubicin	Topoisomerase II poison	DSBR
Topotecan, Irinotecan	Topoisomerase I poison	BER/SSBR, HRR
Busulfan, Melphalan, Temozolomide	Alkylating agent	BER/SSBR

*BER/SSBR, base excision repair/single strand break repair; DSBR, double strand break repair; FA, fanconi anemia; HRR, homologous recombination repair; NER, nucleotide excision repair*.

DNA lesions affecting one strand, such as single strand breaks (SSBs), base deamination, oxidation, methylation or loss, bulky DNA adducts or intra-strand cross links are repaired by base excision repair (BER) (108), SSB repair (SSBR) (109), and nucleotide excision repair (NER) (110). Poly (ADP-ribose) polymerases 1 and 2 (PARP1 and PARP2) signaling is required for efficient SSBR (111) for which many inhibitors have been developed (section Cell Cycle Checkpoint Signaling and Replication Stress).

Errors made during replication, known as mismatches, are repaired by the mismatch repair (MMR) pathway. In this pathway, errors in the newly synthesized strand are removed and the DNA is resynthesized by the replication machinery (112). Bulky abducts which remain unrepaired at DNA replication are bypassed by a process known as translesion synthesis (TLS), which allows restoration of the double stranded DNA prior to NER (113).

DNA damage affecting both DNA strands, such as double strand breaks (DSBs), replication stress and interstrand cross-links are repaired by non-homologous end joining (NHEJ) (114), homologous recombination repair (HRR) (115) and the Fanconi anemia (FA) pathway. The FA pathway is required for the repair of interstrand cross-links and is mediated by the large multimeric FA complex. The TLS, HRR, and NER pathways are also required to repair the DNA after excision of the cross-link from one DNA strand (116).

### DDR Defects in Neuroblastoma

**Figure 4:**
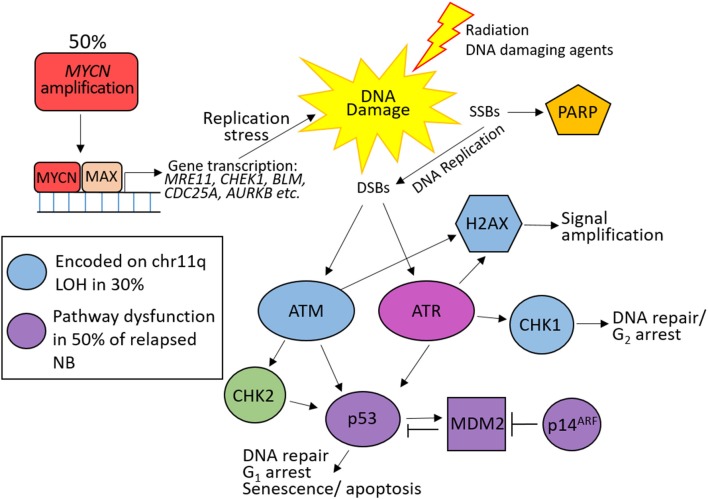
DNA damage response defects in high risk neuroblastoma (HR-NB). 50% of HR-NB are *MYCN* amplified resulting in increased expression of DNA repair genes (*MRE11, CHEK1, BLM* etc.) and genes driving proliferation (*CDC25A, AURKB* etc.), leading to replication stress and DNA damage through replication errors. 30% harbor 11q loss of heterozygosity (LOH) resulting in reduced expression of many proteins involved in the response to double strand breaks (blue). p53 pathway (purple) dysfunction is common at NB relapse leading to defective G_1_ checkpoint arrest.

Figure 4 shows an overview of DDR defects in HR-NB. Among the most common lesion is allelic loss of chromosome 11q. Many DDR proteins are encoded on 11q and are included within the smallest region of overlap, including *ATM, CHK1, MRE11*, and *H2AFX*. Although no mutation or hyper-methylation (silencing) was found in the other allele of these genes in most cases (44), loss of one copy via 11q deletion could result in reduced expression of these proteins, compromising DNA damage signaling and DSB repair, and contributing to replication stress.

Around 50% of HR-NB have an amplification of the *MYCN* oncogene, which drives proliferation and causes replication stress (117). MYCN also transcriptionally upregulates many proteins involved in DNA DSB repair, including components of the MRE11-RAD50-NBLS1 (MRN) complex (118, 119), alternative NHEJ (alt-NHEJ) (120), and Bloom syndrome (BLM) helicase (121), and the cell cycle checkpoint protein CHK1 (117, 122). Upregulation of these genes likely provide MYCN-driven tumors the ability to tolerate higher levels of DNA damage and replication stress. 11q loss is rarely observed in *MYCN*–amplified NB therefore this subset of HR-NB could show benefit from treatments targeting ATM (which is activated by MRN) and/or CHK1.

Loss of G_1_ checkpoint control in NB, through reduced ATM expression, loss of p53 function, and overexpression of MYCN (promotes premature S phase entry and increases replication stress) result in intra-S and G_2_/M checkpoint dependency in these cells, in order to prevent mitosis with damaged DNA, and are therefore especially vulnerable to its inhibition. In addition to *ATM* loss, MYCN induces ATM downregulation by miR-421 (123). Targeting tumor specific DDR defects with PARP and ATR inhibitors in the treatment of HR-NB could potentially increase survival in this risk group.

## Clinical Development of DDR Inhibitors

### PARP Inhibitors

Inhibition of PARP, the enzyme which promotes the repair of DNA single strand breaks, selectively kills cells defective in homologous recombination repair (HRR), e.g., due to *BRCA* mutation. This is due to synthetic lethality when the function of two complementary pathways are inactivated. In normal cells, blocking the repair of SSBs by PARP inhibition will result in a single ended DSB when the DNA replication machinery reaches this lesion, which is then repaired by HRR. Cancer cells defective in HRR cannot repair this break resulting in cell death. Four PARP inhibitors have been approved to date, Lynparza (olaparib, Astra Zeneca), Rubraca (rucaparib, Clovis oncology), and Zejula (niraparib, Tesaro), for the treatment of platinum sensitive ovarian cancer (124) and Talzenna (talazoparib, Pfizer) for the treatment of germline *BRCA* mutated, HER2 negative breast cancer (125). Other PARP inhibitors, veliparib (Abbvie), and pamiparib (BeiGene) are being investigated in clinical trials, with veliparib having advanced to phase 3.

There is accumulating evidence in favor of introducing PARP inhibitors to HR-NB treatment regimens. In 2009, we showed that the PARP inhibitor rucaparib potentiated the cytotoxic effect of temozolomide and topotecan in both *in vitro* and *in vivo* models of NB (126). Subsequent studies have also shown that PARP inhibition increases sensitivity to a variety chemotherapeutic agents and to ionizing radiation in preclinical models of NB (127, 128).

PARP inhibitors have been reported to be synthetically lethal in cells with 11q deletions and *ATM* mutations in lymphoid tumors (129). Recent studies in preclinical models of NB have also shown that 11q loss confers sensitivity to PARP inhibitors (130, 131), further supporting the hypothesis that heterozygous loss of ATM and other DDR genes determines sensitivity to PARP inhibition.

In addition to 11q, Colicchia et al. showed that PARP inhibition enhances replication stress in *MYCN* amplified cells and leads to increased cell death through mitotic catastrophe as these cells enter S-phase with damaged DNA (132). The mechanism suggested in this paper defines another subgroup of HR-NB tumor whereby PARP inhibitors might be beneficial therapeutically.

Early phase international clinical trials are currently testing the efficacy of PARP inhibitors (PARPi) for the treatment of childhood solid tumors with defects in DDR genes, including NB. These are summarized in Table 2.

**Table 2:** PARP inhibitors currently in pediatric clinical trials.

**Inhibitor**	**Company**	**In combination with**	**Disease**	**Trial phase**	**Trial number**	**Status**
Olaparib	Astra Zeneca	N/A	Relapsed or refractory solid tumors^*^, non-Hodgkin lymphoma, or histiocytic disorders with defects in DDR genes	2	NCT03233204	Recruiting
		Irinotecan	Relapsed or refractory tumors with molecular abnormities^*^	1/2	NCT02813135	Recruiting
Talazparib	Pfizer	Irinotecan with or without temozolomide	Refractory or recurrent solid tumors	1	NCT02392793	Active, not recruiting
Veliparib	AbbVie	Temozolomide	Refractory or recurrent CNS tumors	1	NCT00994071	Completed

* Including neuroblastoma; N/A, not applicable.

*Information from clinicaltrial.gov (February, 2020)*.

However, it is worth noting that the combination of PARPi with conventional chemotherapy in adults leads to increased hematological toxicity (133), with doses of the PARP inhibitor and cytotoxic chemotherapy combination subsequently being reduced. This observation is reflected in the results of a pediatric trial combining veliparib (ABT-888) with temozolomide in brain tumors (134), where the main dose limiting toxicity was myelosuppression. In the case of NB (and other pediatric tumors), a reduction in chemotherapy doses when combined with a PARP inhibitor might be advantageous in reducing the long term toxicity of these drugs, if efficacy is maintained.

### ATR Inhibitors

Replication stress and defects in G_1_ cell cycle control render cells highly dependent on ATR and hence should be sensitive to its inhibition (135). Four inhibitors of ATR are now in clinical trials: M6620 (berzosertib, formally VX-970, Merck), M4344 (formally VX-803, Merck), AZD6738 (ceralasertib, Astra Zeneca), and BAY1895344 (Bayer) (Table 3).

**Table 3:** ATR inhibitors currently in adult clinical trials.

**Inhibitor**	**In combination with**	**Trial phase**	**Disease**	**Trial numbers**
M6620/VX-970/berzosertib (Merck)	Irinotecan	1	Metastatic solid tumors	NCT02595931
	Cisplatin and radiotherapy	1	Head and neck squamous cell carcinoma	NCT02567422
	Radiotherapy	1	Chemotherapy resistant breast cancer	NCT04052555
	Cisplatin, capecitabine, radiotherapy	1	Solid tumors	NCT03641547
	Carboplatin and paclitaxel	1	Advanced solid tumors	NCT03309150
	Carboplatin and Avelumab	1/2	PARPi-resistant ovarian cancer	NCT03704467
	Topotecan	1/2	Small cell cancers and extrapulmonary small cell cancers	NCT02487095
	N/A	2	Selected solid tumors	NCT03718091
	Topotecan	2	Relapsed or extrapulmonary small cell lung cancer	NCT03896503
	Irinotecan	2	Progressive, metastatic, or unresectable *TP53* mutant gastric or gastroesophageal junction cancer	NCT03641313
	Gemcitabine	2	Recurrent ovarian, primary peritoneal, or fallopian tube cancer	NCT02595892
	Cisplatin and gemcitabine	2	Metastatic urothelial cancer	NCT02567409
	Carboplatin	2	Metastatic castrate-resistant prostate cancer	NCT03517969
M4344/ VX-803 (Merck)	Carboplatin, gemcitabine or cisplatin	1	Advanced solid tumors	NCT02278250
AZD6738/ceralasertib (Astra Zeneca)	N/A	1	Myelodysplastic Syndrome or Chronic Myelomonocytic Leukemia	NCT03770429
	Gemcitabine	1	Inoperable/unresectable, locally advanced or metastatic solid tumor that has progressed on standard therapy	NCT03669601
	Radiotherapy	1	Refractory solid tumor	NCT02223923
	Paclitaxel	1	Refractory cancer	NCT02630199
	Olaparib	1	Head and neck squamous cell carcinoma	NCT03022409
	Acalabrutinib	1	relapsed/refractory aggressive Non-Hodgkin's Lymphoma	NCT03527147
		1/2	Chronic lymphocytic leukemia	NCT03328273
	Olaparib, durvalumab (PD-L1 antibody), or carboplatin	1/2	Advanced solid tumors	NCT02264678
	Olaparib	2	Metastatic Triple Negative Breast Cancer	NCT03330847
		2	Ovarian high grade serous carcinoma	NCT03462342
		2	Renal cell carcinoma, urothelial carcinoma, pancreatic ductal adenocarcinoma, or other metastatic solid tumors	NCT03682289
		2	Isocitrate dehydrogenase (IDH) 1 or 2 mutant tumors	NCT03878095
		2	Relapsed small cell lung cancer	NCT03428607
		2	Resistant prostate cancer	NCT03787680
		2	Metastatic castrate resistance prostate cancer	NCT02576444
		2	Gynecological cancers	NCT04065269
	Durvalumab	2	Gastric adenocarcinoma and malignant melanoma	NCT03780608
		2	Non-small cell lung cancer	NCT03334617
		2	Non-small cell lung cancer with PD-1 immune checkpoint inhibitor resistance	NCT03833440
BAY-1895344 (Bayer)	N/A	1	Advanced solid tumors and lymphomas	NCT03188965

*Information from clinicaltrial.gov (February, 2020); N/A, not applicable*.

Both amplification of *MYCN* and impaired ATM function, which result in replication stress and defects in G_1_ cell cycle control, are known determinants of sensitivity to ATR inhibitors (105, 136). There is some evidence that chemosensitization by ATR inhibitors relies on a dysfunctional p53 pathway, and therefore a defective G_1_/S checkpoint (105, 137). p53 pathway dysfunction is rare in NB at diagnosis but frequent abnormalities are observed at relapse (59). Collectively, *MYCN* amplification and allelic 11q deletion are observed in 70–80% of HR-NB tumors (44), suggesting a large group of HR-NB patients may benefit from treatment with ATR inhibitors.

Inhibition of ATR has been shown to mediate sensitivity to PARP inhibition (138, 139). PARP inhibition results in DNA DSBs in S-phase, which require activity of ATR signaling to S phase cell cycle arrest and HRR for repair. ATR inhibition has also been shown to overcome acquired resistance to PARP inhibitors (140, 141). In theory, the combination with ATR inhibitors should potentiate the cytotoxic effects of PARP inhibitors in the treatment of NB.

It has been suggested that cancer cells which maintain their telomeres by alternative lengthening have increased sensitivity to ATR inhibition (142). ALT is found in 50% of NB cells which harbor loss of function mutations or intragenic deletions in *ATRX* (74, 75)*. ATRX* mutation has been observed in around 25% of HR-NB (73). However, it has subsequently been reported that ALT is not an independent determinant of ATR inhibitor sensitivity (143). At present, it is unclear whether *ATRX* loss of function will define a subset of NB cells sensitive to ATR inhibition due to telomere maintenance by ALT.

The efficacy of the ATR inhibitor clinical candidate from Merck, M6620 (formerly VX-970, Vertex), has recently been tested alone and in combination with cisplatin and melphalan in a range of pediatric solid tumor cell lines and xenograft models including NB (144). This study showed that M6620 had limited single agent cytotoxicity but potentiated the cytotoxic effects of cisplatin and melphalan in the majority of cell lines tested. Although limited, this study indicated that ATR inhibitors could potentially be beneficial when used in combination with existing chemotherapeutic regimens.

Further studies are required to determine which molecular abnormalities confer sensitivity to ATR inhibition in NB.

### CHK1 Inhibitors

CHK1 kinase is the direct downstream effector of ATR. *MYCN* amplified NB cell lines show sensitivity to CHK1 inhibitors as single agents (145, 146) and as a chemosensitizer to cytotoxic agents (145, 147). Although CHK1 inhibitors have been in clinical development for many years, many compounds have been discontinued before Phase 3 trials due to toxicities. Two CHK1 inhibitors, Prexasertib (LY2606368; Eli Lilley) and SRA737 (Sierra Oncology) are currently being tested in clinical trials, with Prexasertib having entered a Phase 1 clinical trial in pediatric solid tumors (NCT0280865, NCT04023669).

### WEE1 Inhibition

WEE1 is a key kinase in the activation of the S and G_2_/M cell cycle checkpoints in response to DNA damage. Phosphorylation of CDK1 by WEE1 keeps CDK1 in an inactive state, thus preventing entry into mitosis (148). Single agent treatment with the WEE1 inhibitor Adavosertib was shown to be effective in both *in vitro* and *in vivo* preclinical NB models (145). In the same study, Adavosertib was shown to be synergistic with the CHK1 inhibitor MK-8776, the topoisomerase I poison SN-38 (active metabolite of irinotecan) and gemcitabine.

Adavosertib is currently the only WEE1 inhibitor in clinical development and has advanced into Phase II clinical trials for the treatment of pediatric solid tumors including NB (Table 4).

**Table 4:** Current pediatric clinical trials of the WEE1 inhibitor Adavosertib.

**Inhibitor**	**Company**	**In combination with**	**Disease**	**Trial phase**	**NCT number**
Adavosertib (AZD1775/MK-1775)	Astra Zeneca	Local radiation	DIPG	1	NCT01922076
		Irinotecan	Relapsed or refractory solid tumors	1/2	NCT02095132
		Carboplatin	Relapsed or refractory solid tumors with molecular abnormities	1/2	NCT02813135

DIPG, diffuse intrinsic pontine glioma.

*Information from clinicaltrial.gov (February, 2020)*.

### ATR and PARP Inhibitor Combinations in the Clinic

Since ATR inhibition has been shown to overcome PARPi resistance by abrogating the G_2_ checkpoint, there are many clinical trials testing this combination. PARP inhibition increases replication stress (132), which would also render cells dependent on ATR inhibition. A summary of currently listed clinical trials involving a combination of PARP and ATR inhibitors is listed in Table 5.

**Table 5:** ATR and PARP inhibitor combinations in adult clinical trials.

**ATR inhibitor**	**PARP inhibitor**	**Cancer type**	**Phase**	**Trail number**	**Status**
M6620 (VX-970)	Veliparib (+cisplatin)	Refractory solid tumors	1	NCT02723864	Recruiting
AZD6738	Olaparib	Head and neck squamous cell carcinoma (HNSCC)	1	NCT03022409	Recruiting
		Advanced solid malignancies—HNSCC, non-small cell lung cancer, gastric and breast cancer	1/2	NCT02264678	Recruiting
		Ovarian high grade serous carcinoma	2	NCT03462342	Recruiting
		Patients with tumors harboring mutations in homologous DNA repair genes, including *ATM, CHK2, APOBEC, MRE11* complex	2	NCT02576444	Recruiting
		Metastatic triple negative breast cancer with alterations in HRR genes	2	NCT03330847	Recruiting
		Renal cell carcinoma, urothelial carcinoma, pancreatic ductal adenocarcinoma, or other metastatic solid tumors	2	NCT03682289	Not yet recruiting
		Relapsed small cell lung cancer	2	NCT03428607	Not yet recruiting
		Resistant prostate cancer	2	NCT03787680	Not yet recruiting
		Metastatic castrate resistance prostate cancer	2	NCT02576444	Not yet recruiting
		Gynecological cancers	2	NCT04065269	Not yet recruiting
		Isocitrate dehydrogenase (IDH) 1 or 2 mutant tumors	2	NCT03878095	Not yet recruiting

*Information from clinicaltrial.gov (February, 2020)*.

## Conclusion

Improving survival rates for HR-NB remains a challenge in pediatric oncology. If long term survival is achieved, high risk patients are often left with severe sequelae as a result of high dose chemotherapy, and relapse is common. Many features of HR-NB suggest that subsets of these tumors will be sensitive to DDR inhibitors. Mutation or loss of genes such as *ATM* or others involved in HRR suggest sensitivity to PARP inhibition. Around half of HR-NB tumors are *MYCN*-amplified, which would lead to sensitivity to ATR inhibition due to oncogene induced replication stress. In addition, the frequent loss of G_1_ checkpoint control in HR-NB, by *MYCN* amplification and p53 pathway loss at relapse, provide a rationale for treatment with G_2_ checkpoint targeting agents (ATR, CHK1, and/or WEE1 inhibitors). Further work to identify predictive biomarkers of sensitivity to DDR inhibitors in NB will better stratify patients who might benefit from these agents.

As well as single agent efficacy of DDR inhibitors, there is mounting evidence to suggest that combining these agents with conventional chemotherapeutics or radiotherapy would permit lower doses to be given with the same effect due to chemo- and radio-sensitization.

Although inhibitors of these target proteins have been in adult trials for many years, the potential for their use in the treatment of pediatric tumors has only recently been explored. It is worth noting that even though a compound is effective in adult cancers, the same may not be true in the pediatric setting. It is also important to consider the potential long term toxicity of inhibiting the DDR, such as the development of secondary malignancies. Although unlikely to cause more off-target effects than current high dose chemotherapy regimens, the long-term toxicity of these agents in children is unknown and may take years to become apparent.

Nevertheless, exploiting defects in the DDR has the potential to lead to novel therapeutic options for a large subset of HR-NB patients for whom the prognosis is still unacceptably poor.

## Author Contributions

HS collected the data and wrote the manuscript. HS, LC, NC, and DT contributed to the design and content of the review, read, and approved the final manuscript for publication.

### Conflict of Interest

NC has received research funding from Aguoron Pharmaceuticals, Pfizer and Clovis Oncology for the development of rucaparib, BioMarin for studies on talazoparib and Tesaro for studies with niraparib and paid consultancy from Abbvie for veliparib. NC is inventor on the patent relating to the use of rucaparib in HRD cancer (WO2005012305A3) and Newcastle University receives royalty income from the sales of rucaparib that are shared with contributors to its development, including NC. NC does not take these royalties personally. NC has also received research funding from Vertex Pharmaceuticals and is currently in receipt of funding from Merck KGaA for work on ATR inhibitors and holds a patent for a pharmacodynamic biomarker of ATR inhibition (WO2014055756A1). The remaining authors declare that the research was conducted in the absence of any commercial or financial relationships that could be construed as a potential conflict of interest.
